# Exploratory Gene Expression Profiling of Cisplatin-Induced Neurotoxicity in Rat Brain

**DOI:** 10.3390/ijms262110299

**Published:** 2025-10-23

**Authors:** Osvaldo Torres-Pineda, Consuelo Morgado-Valle, Donají Chi-Castañeda, María Leonor López-Meraz, Christian Martin Rodríguez-Razón, Monserrat Macías-Carballo, Luis Beltrán-Parrazal

**Affiliations:** 1 Instituto de Investigaciones Cerebrales, Universidad Veracruzana, Xalapa 91190, Veracruz, Mexico; osvaldo.torres.pineda@gmail.com (O.T.-P.); comorgado@uv.mx (C.M.-V.); lchi@uv.mx (D.C.-C.); leonorlopez@uv.mx (M.L.L.-M.); 2 Departamento de Ciencias de la Salud, University Center of Los Altos, University of Guadalajara, Tepatitlán de Morelos 47620, Jalisco, Mexico; christian.rrazon@academicos.udg.mx

**Keywords:** cisplatin, neurotoxicity, chemotherapy-related cognitive impairment (CRCI), transcriptomics, gene expression, neuroinflammation, pathway analysis

## Abstract

Cisplatin is a widely used antineoplastic agent whose therapeutic efficacy is often limited by its adverse effects on the central nervous system. In this exploratory study, we characterized the transcriptomic impact of a cumulative cisplatin regimen on the male Wistar rat brain using microarray technology. Differentially expressed genes were identified, and their functional roles were investigated through enrichment analyses (KEGG) and Gene Ontology (GO), and the construction of protein–protein interaction (PPI) networks. Our results revealed significant alterations in pathways related to synaptic signaling, neuroplasticity, and cellular metabolism. To generate translational hypotheses, these findings were subsequently correlated in silico with public human lower-grade glioma (LGG) datasets, which suggested a potential association between key cisplatin-regulated genes and clinical prognosis and immune cell infiltration patterns. This manuscript does not include RT-qPCR (or Western blot) validation; results should be interpreted as hypothesis-generating and require orthogonal confirmation. These findings provide a comprehensive transcriptomic map of cisplatin-induced neurotoxicity, offering novel insights into its underlying molecular mechanisms and identifying a rich set of candidate targets for future neuroprotective strategies.

## 1. Introduction

Cisplatin is a widely used chemotherapeutic agent for the treatment of various solid tumors, including lung, ovarian, bladder, and testicular cancers [1,2]. Its antitumor mechanism relies on the formation of DNA cross-links, interfering with replication and transcription processes, which leads to the activation of pro-apoptotic pathways [2]. However, its effectiveness is limited by the emergence of severe side effects, including neurotoxicity that affects both the peripheral and central nervous systems [1,3].

Clinical evidence has shown that a significant proportion of patients treated with cisplatin develop persistent cognitive impairment, known as “chemo-brain” or chemotherapy-induced cognitive dysfunction, which includes deficits in attention, memory, and processing speed. These alterations have been corroborated by neuroimaging studies revealing structural changes in the prefrontal cortex, hippocampus, and white matter [1,4].

Murine models have enabled the characterization of cisplatin’s effects on the central nervous system (CNS), demonstrating that its administration results in mitochondrial dysfunction and alterations in synaptic integrity [1,5]. These effects are mediated by a cascade of molecular events, including the activation of signaling pathways such as MAPK, NF-κB, p53, and the antioxidant response regulated by NFEL2 (*NRF2*) [6,7,8]. Furthermore, the overexpression of genes associated with inflammation, autophagy, oxidative damage, and energy dysfunction has been documented [6,7]. In this context, transcriptomics has emerged as a key tool to understand the systemic effects of neurotoxic agents. Microarray analyses allow the simultaneous quantification of the expression of thousands of genes via hybridization-based detection, facilitating the identification of differential molecular profiles induced by pharmacological treatments [6,8]. In the case of cisplatin, previous transcriptomic studies have reported alterations in biological pathways related to synaptic plasticity, apoptosis, mitochondrial metabolism, and epigenetic regulation [5,9,10]. Nevertheless, most of these studies have focused on tumor cell lines or peripheral tissues, leaving a significant gap in our understanding of cisplatin’s effects on the non-tumoral brain transcriptome in vivo [9,11]. To address this gap, the present study characterizes the transcriptomic profile of the brain of male Wistar rats exposed to a cumulative cisplatin regimen using microarray analysis. Our aim is to identify differentially expressed genes and their associated biological pathways to elucidate the molecular mechanisms underlying cisplatin-induced central neurotoxicity. The findings from this exploratory transcriptomic approach may reveal novel insights and contribute to the design of future therapeutic strategies to mitigate the adverse neurological effects of this widely used chemotherapy.

## 2. Results

### 2.1. Identification of Differentially Expressed Genes and Comparative Analysis

The microarray analysis identified a total of 143 genes as significantly upregulated (Z-score ≥ 2) and 163 genes as significantly downregulated (Z-score ≤ −2) in the brain tissue of cisplatin-treated rats compared to controls.

**Figure 1:**
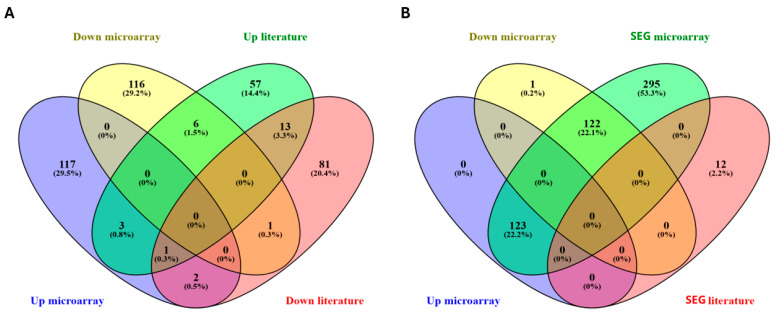
Venn diagrams showing the overlap between differentially expressed genes and sets from the literature or those with stable expression. (**A**) Intersection between upregulated (Z ≥ 2) and downregulated (Z ≤ −2) genes identified by microarray with gene sets reported in the literature as upregulated or downregulated. (**B**) Venn diagrams. Differentially expressed genes (DEG) and stably expressed genes (SEG) according to the literature under control conditions [12,13]. UBB, BCL2, and FAS are highlighted as SEG, not differentially expressed in our dataset. Percentages reflect the proportion of genes within each category relative to the total combined set.

To contextualize these findings, we first compared our results with a curated database of genes previously reported as dysregulated by cisplatin in other models (Figure 1A). This revealed several genes with discordant regulation; for instance, six genes downregulated in our dataset (*PRKCZ*, *GRPR*, *PLA2G2A*, *PLCB4*, *SARDH*, and *GABRR1*) were previously reported as upregulated. Conversely, two genes upregulated in our study (*HOXA5* and *NFE2L2*) were reported as downregulated elsewhere. However, we also found consistent patterns: three upregulated genes (*CAMK4*, *FANCC*, and *GRIA4*), known to be involved in synaptic signaling and DNA repair pathways, and one downregulated gene (*ABCC2*), were regulated in the same direction as in prior reports. In contrast, twelve genes reported in the literature as stably expressed under control conditions—including UBB, BCL2, and FAS—were not detected as differentially expressed in our experiment [12,13]. Notably, *CDK4*, a key cell cycle regulator, was found to be upregulated in our data and has been reported with context-dependent up- or downregulation in the literature.

A second analysis evaluated the overlap between the differentially expressed genes and genes exhibiting stable basal expression in our control group, which we defined as those with Z-score values between −1.5 and 1.5 (Figure 1B). This analysis showed a substantial overlap, with 123 upregulated and 122 downregulated genes falling within this stable expression range under basal conditions, suggesting that cisplatin significantly alters genes that are otherwise tightly regulated. Among these, *CAMK4*, *FANCC*, *GRIA4*, and *NFE2L2* were also highlighted in the literature comparison, reinforcing their potential relevance.

Finally, the analysis identified specific genes unique to certain categories. For example, the gene *SLC1A3* was the only gene downregulated in our dataset with no other overlaps. In contrast, twelve genes reported in the literature as stably expressed under control conditions (SEG)—including UBB, BCL2, and FAS—were not detected as differentially expressed in our experiment [12,13]

### 2.2. Kyoto Encyclopedia of Genes and Genomes (KEGG) Pathway Enrichment Analysis

To understand the functional implications of the observed gene expression changes, we performed a KEGG pathway enrichment analysis for the up- and downregulated gene sets.

**Figure 2:**
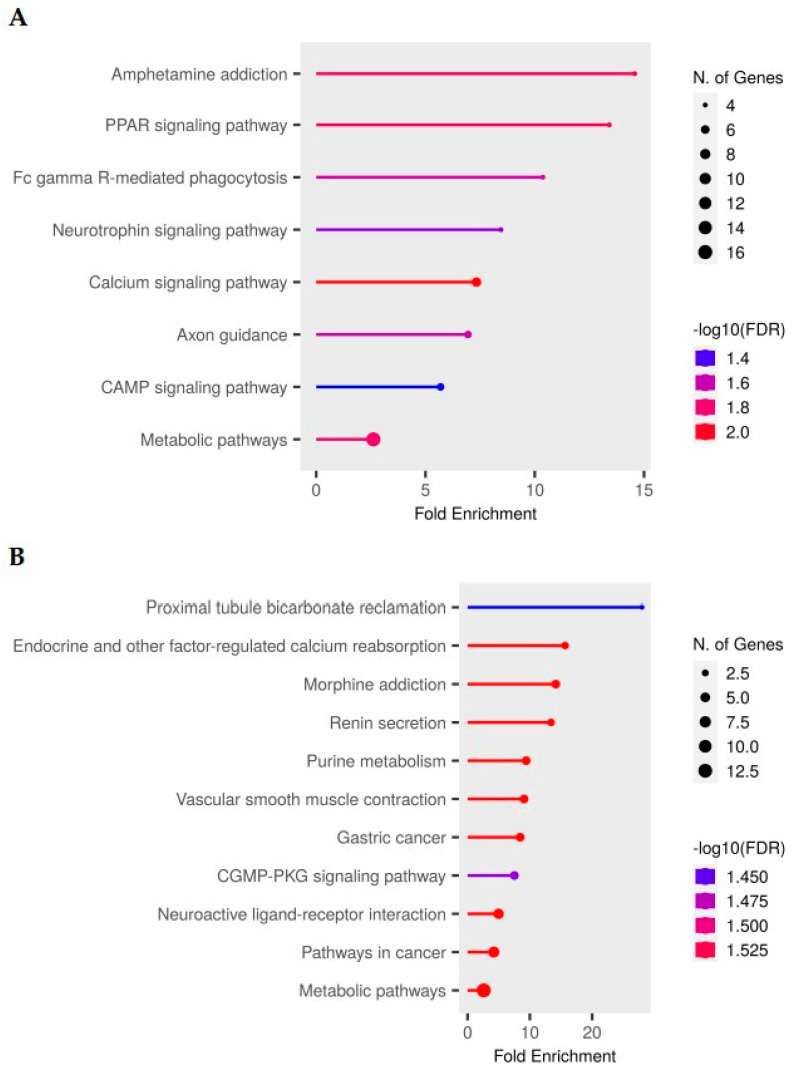
KEGG pathway enrichment analysis of differentially expressed genes following cisplatin treatment. (**A**) Pathways enriched in upregulated genes (Z ≥ 2). (**B**) Pathways enriched in downregulated genes (Z ≤ −2). Pathways are ranked by false discovery rate (FDR), with rightward position indicating greater fold enrichment. Dot color represents −log10(FDR), and dot size indicates the number of genes in each pathway. Complete KEGG/GO pathway lists (name, ID, gene count, fold enrichment, raw P, FDR) are provided in Supplementary Table S1 (significant at FDR ≤ 0.05; suggestive at 0.05–0.10).

The analysis revealed that upregulated genes were predominantly associated with neural signaling and plasticity (Figure 2A). The most prominently enriched KEGG pathways included Amphetamine addiction (KEGG: rno05031), Neurotrophin signaling pathway (KEGG: rno04722), PPAR signaling pathway (KEGG: rno03320), and Axon guidance (KEGG: rno04360). In line with the cross-pathway analysis introduced below, we also note involvement of the calcium signaling pathway (KEGG: rno04020), consistent with activity-dependent transmission.

Conversely, downregulated genes were enriched in homeostatic and metabolic processes (Figure 2B), including Proximal tubule bicarbonate reclamation (KEGG: rno04964), Endocrine and other factor-regulated calcium reabsorption (KEGG: rno04961), and Purine metabolism (KEGG: rno00230). Although classically described in renal or systemic contexts, the enrichment of these modules in brain tissue likely reflects dysregulation of shared molecular components—such as solute carriers, ATPases, and calcium-handling proteins—also critical for neuronal signaling and neurovascular regulation. Suggestively, Renin secretion (KEGG: rno04924) and Vascular smooth muscle contraction (KEGG: rno04270) were also observed (FDR ≈ 0.067).

Additional significantly enriched KEGG pathways (BH-FDR ≤ 0.05; official names and IDs) are:

Upregulated: Amphetamine addiction (KEGG: rno05031)—4 genes, FDR = 0.0094; PPAR signaling pathway (KEGG: rno03320)—4 genes, FDR = 0.0100; Fc gamma R–mediated phagocytosis (KEGG: rno04666)—4 genes, FDR = 0.0210; Axon guidance (KEGG: rno04360)—5 genes, FDR = 0.0220; Neurotrophin signaling pathway (KEGG: rno04722)—4 genes, FDR = 0.0320; Human immunodeficiency virus 1 infection (KEGG: rno05170)—5 genes, FDR = 0.0320; cAMP signaling pathway (KEGG: rno04024)—5 genes, FDR = 0.0390; Metabolic pathways (KEGG: rno01100)—17 genes, FDR = 0.0081.

Downregulated: Proximal tubule bicarbonate reclamation (KEGG: rno04964)—2 genes, FDR = 0.046; Hepatitis B (KEGG: rno05161)—3 genes, FDR = 0.024; Endocrine and other factor-regulated calcium reabsorption (KEGG: rno04961)—3 genes, FDR = 0.025; Morphine addiction (KEGG: rno05032)—4 genes, FDR = 0.014; Purine metabolism (KEGG: rno00230)—4 genes, FDR = 0.029; Gastric cancer (KEGG: rno05226)—4 genes, FDR = 0.044; cGMP–PKG signaling pathway (KEGG: rno04022)—4 genes, FDR = 0.046; Pathways in cancer (KEGG: rno05200)—7 genes, FDR = 0.046; Metabolic pathways (KEGG: rno01100)—13 genes, FDR = 0.046.

Suggestive (0.05 < FDR ≤ 0.10; listed in Supplementary Table S1): Renin secretion (KEGG: rno04924)—4 genes, FDR = 0.067; Vascular smooth muscle contraction (KEGG: rno04270)—5 genes, FDR = 0.067; Neuroactive ligand–receptor interaction (KEGG: rno04080)—8 genes, FDR = 0.067.

To complement the upregulated-gene convergence, we examined overlaps between downregulated GO Biological Process categories and KEGG pathways. Three consistent axes emerged. First, GO clusters related to response to external stimulus and regulation of ion and molecular transport overlapped with the KEGG Neuroactive ligand–receptor interaction pathway (KEGG: rno04080; suggestive, FDR ≈ 0.067), with representative downregulated receptors including *GABRA1*, *CHRM1*, *TRHR*, *GRPR*, and *ADORA1*, consistent with reduced neuromodulator receptor signaling. Second, ion-handling GO terms converged with significant KEGG modules Proximal tubule bicarbonate reclamation (rno04964) and Endocrine and other factor-regulated calcium reabsorption (rno04961), indicating a coordinated decrease in solute-carrier/ATPase–mediated transport and Ca^2+^ homeostasis. Third, downregulation of *PDE7A* links GO signal-transduction clusters to significant KEGG Purine metabolism (rno00230), implicating diminished second-messenger turnover (cAMP/cGMP). Together, these overlaps suggest that cisplatin suppresses neuromodulator receptor signaling and ion-transport/nucleotide-metabolism modules, potentially weakening synaptic modulation and homeostatic resilience in brain tissue. Complete pathway names/IDs, gene counts, and FDR values are provided in Supplementary Table S1 (significant at FDR ≤ 0.05; suggestive at 0.05–0.10).

#### Cross-Pathway Gene Analysis: GO–KEGG Convergence

To identify molecular nodes linking GO synaptic processes with KEGG calcium signaling, we intersected the genes driving synaptic signaling (GO:0099536) and anterograde trans-synaptic signaling (GO:0098916) with KEGG calcium signaling (KEGG:rno04020). Among upregulated genes, we identified shared components including *CAMK4*, *PLCG1*, *GRIA4*, *CACNA1E*, and *ADORA2A*. These hubs connect presynaptic and postsynaptic transmission to calcium-dependent cascades (CaMK, PLC/IP3/DAG, voltage-gated Ca^2+^ influx, and cAMP–Ca^2+^ crosstalk), supporting a model in which cisplatin engages plasticity-related signaling via Ca^2+^ homeostasis and activity-dependent transcription.

### 2.3. Network Analysis of Enriched GO Biological Processes

To visualize the complex interplay between the biological functions affected by cisplatin, we constructed interaction networks based on enriched Gene Ontology (GO) Biological Process terms.

**Figure 3:**
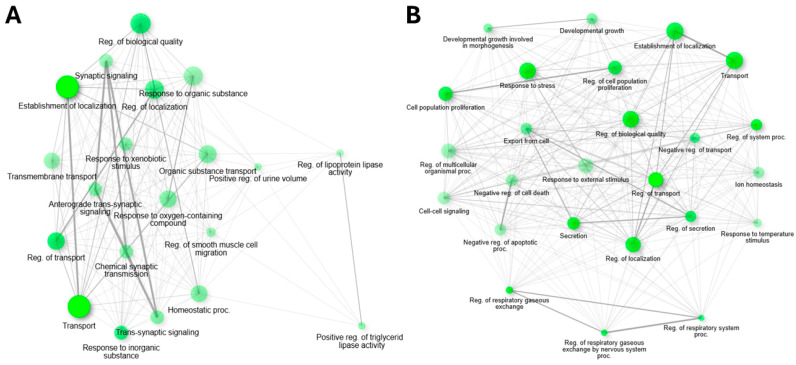
Functional network analysis based on enriched GO Biological Process terms. (**A**) Network of upregulated genes (Z ≥ 2). (**B**) Network of downregulated genes (Z ≤ −2). Each node represents an enriched GO term, with node size proportional to the number of associated genes. Connections indicate shared genes between terms, with line thickness reflecting the degree of gene overlap.

The network for upregulated genes (Figure 3A) revealed a highly interconnected cluster of processes centered on neuronal communication. The most prominent node was synaptic signaling (GO:0099536), which showed strong connections to related terms such as chemical synaptic transmission (GO:0007268) and anterograde trans-synaptic signaling (GO:0098916). This highlights the central role of synaptic plasticity in the brain’s response to cisplatin. Other important connected processes included “regulation of transport” and “response to xenobiotic stimulus”.

In contrast, the network of downregulated genes (Figure 3B) displayed a more distributed topology with several distinct but interconnected clusters, suggesting a broad disruption of core cellular functions. Key clusters were associated with regulation of cell population proliferation (GO:0042127), developmental growth (GO:0048589), response to external stimulus (GO:0009605), and the regulation of ion and molecular transport (GO:0060341) categories. The dense connectivity pattern suggests a widespread dysregulation of homeostatic processes in brain tissue following treatment.

In both networks, each node represents an enriched GO term, with its size corresponding to the number of associated genes and its color intensity indicating the statistical significance (FDR). The edges between nodes reflect the degree of gene overlap, revealing the functional relationships between these biological processes. This analysis identifies the key functional modules that are differentially impacted by cisplatin-induced up- and downregulation.

### 2.4. Visualization of Key KEGG Pathways

For visualization, we selected two neurofunctionally relevant pathways—one significant and one suggestive—namely the calcium signaling pathway (FDR < 0.05) and the neuroactive ligand–receptor interaction pathway (FDR ≈ 0.067), to highlight complementary aspects of transmitter-receptor coupling and second-messenger cascades.

**Figure 4:**
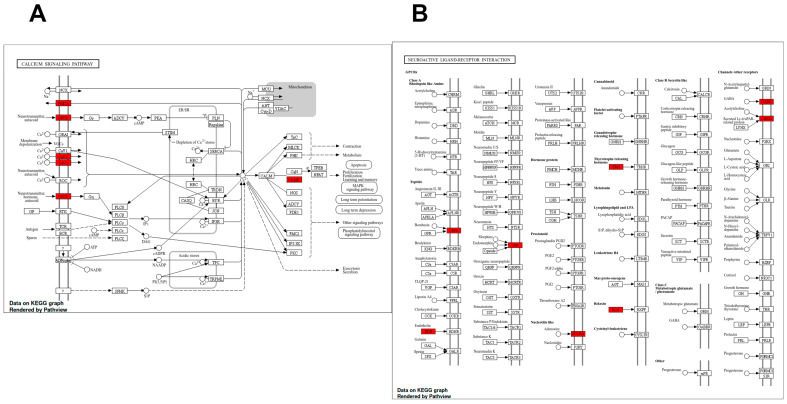
KEGG pathway maps showing differentially expressed genes following cisplatin treatment. (**A**) Calcium signaling pathway highlighting upregulated genes (Z ≥ 2), including *CAMK*, *GPCR*, *PLCB*, and voltage-dependent calcium channel subunits (CaV1, CaV2, CaV3). (**B**) Neuroactive ligand-receptor interaction pathway highlighting downregulated genes (Z ≤ −2), including *GABRA1*, *CHRM1*, *TRHR*, and *GRPR*. Red-highlighted genes indicate differential expression identified by microarray analysis.

In the calcium signaling pathway (Figure 4A), several key upregulated genes were identified, including *CAMK4*, *PLCG1*, and subunits of voltage-dependent calcium channels such as *CACNA1E*. These elements are critical for intracellular calcium mobilization and are known to play essential roles in synaptic plasticity, apoptosis, and neuronal signal transduction.

In the neuroactive ligand-receptor interaction pathway (Figure 4B), we observed the downregulation of multiple genes central to synaptic communication, such as *GABRA1*, *CHRM1*, *TRHR*, and *GRPR*. These genes encode receptors for major neurotransmitters and neuropeptides, and their coordinated downregulation suggests a potent disruption of synaptic transmission mechanisms in response to cisplatin.

These pathway representations illustrate how the specific gene expression changes induced by cisplatin may converge to impact critical homeostatic and signaling networks in the brain.

### 2.5. Key Upregulated and Downregulated Genes in Neurofunctional Pathways

**Figure 5:**
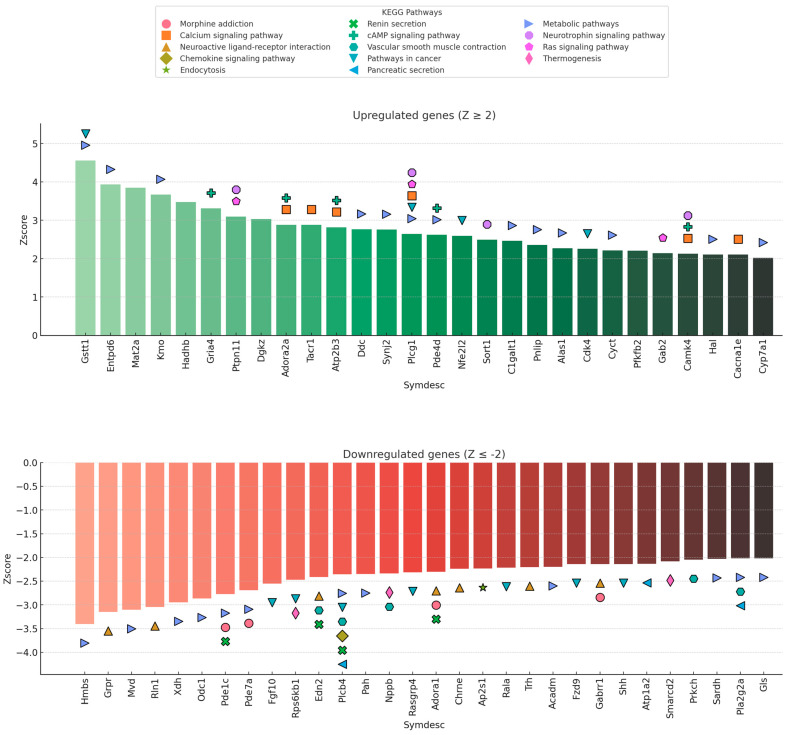
Representative upregulated (Z ≥ 2) and downregulated (Z ≤ −2) genes and their association with KEGG-enriched pathways. Genes are ordered by Z-score intensity, with each bar representing individual gene expression changes. Colored symbols **above** (upregulated) or **below** (downregulated) bars indicate involvement in specific functional pathways. Symbol shape and color correspond to KEGG pathways as shown in the legend.

Our gene expression analysis revealed the differential regulation of multiple genes integral to neuronal homeostasis. Figure 5 highlights the most prominent of these genes based on their Z-score values, illustrating their involvement in the KEGG pathways identified previously. A comprehensive list, including their biological functions, is available in Table 1.

Among the top upregulated genes, *PLCG1*, *CAMK4*, *ADORA2A*, and *GRIA4* were particularly noteworthy. These genes participate in pathways such as the calcium, neurotrophin, and cAMP signaling pathways, which point toward the activation of adaptive or damage-response molecular programs in the brain following cisplatin exposure.

Conversely, the most significantly downregulated genes included *ADORA1*, *GABRR1*, *PLCB4*, *PDE7A*, and *PLA2G2A*. These genes are associated with key pathways like neuroactive ligand-receptor interaction, vascular smooth muscle contraction, and inflammatory signaling. Their coordinated downregulation strongly suggests a disruption of synaptic modulation, neuro-immune communication, and metabolic support within the neural tissue.

### 2.6. Protein–Protein Interaction Networks Reveal Coordinated Functional Modules

To understand the functional relationships between the differentially expressed genes, we constructed Protein–Protein Interaction (PPI) networks for both the up- and downregulated gene sets using the STRING database [14].

**Figure 6:**
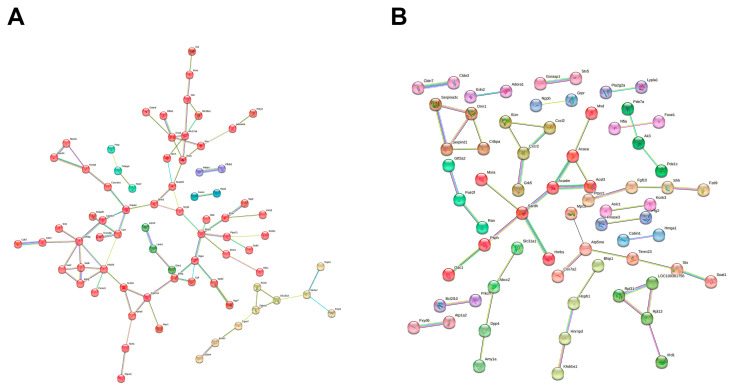
Protein–protein interaction (PPI) networks of differentially expressed genes identified using the STRING database. (**A**) Network of upregulated genes (Z ≥ 2). The network is organized into 6 functional clusters as determined by k-means analysis. (**B**) Network of downregulated genes (Z ≤ −2), organized into 21 functional clusters. Both networks display high-confidence interactions (minimum interaction score ≥ 0.7). Each node represents a protein encoded by a differentially expressed gene, and the node colors denote the functional cluster automatically assigned by STRING.

The network derived from the 143 upregulated genes (Figure 6A) showed strong interconnectivity, which was organized into six main functional clusters via k-means analysis. The largest module (Cluster 1) comprised 55 proteins implicated in neurotransmission, calcium signaling, and metabolic and apoptotic regulation, with key hub nodes including *PLCG1*, *CAMK4*, *ADORA2A*, *GRIA4*, *CACNA1E*, and *CDK4*. Other significant clusters were involved in membrane trafficking (Cluster 2), the Fanconi anemia DNA repair pathway (Cluster 5), and glycolysis regulation via PFKFB3 (Cluster 6). These modules suggest that cisplatin triggers a coordinated adaptive response in the brain, involving the activation of neuroprotective, metabolic compensation, and stress-response pathways.

The network for the 163 downregulated genes (Figure 6B) also revealed a highly connected topology, which k-means analysis segregated into 21 distinct clusters. This modular organization points to a coordinated suppression of key biological processes. Among the most prominent clusters were those involved in fatty acid metabolism (Cluster 1), mitochondrial function (Cluster 2), and *Cxc* chemokine signaling (Cluster 5). Notably, the downregulation of Cluster 6, which includes the heat shock protein *HSPB1*, suggests a compromised protein folding and stress-response capacity. Collectively, this network highlights a broad disruption of pathways related to cellular energy metabolism, neuroinflammation, and proteostasis, potentially reducing the brain’s neuroprotective capabilities.

### 2.7. Correlation of HUB Gene Signatures with Clinical Outcomes in Glioma

To evaluate the prognostic potential of differentially regulated genes under cisplatin treatment, a subset of HUB genes was selected and analyzed for overall survival using the GEPIA2 platform.

**Figure 7:**
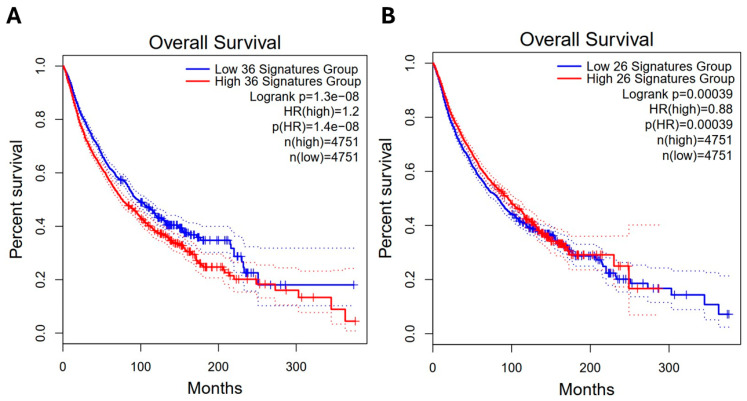
Clinical relevance of cisplatin-regulated gene signatures. (**A**) High expression of 36 upregulated genes (Z ≥ 2) predicts reduced overall survival (Log-rank *p* = 1.3 × 10^−8^; HR = 1.2). (**B**) High expression of 26 downregulated genes (Z ≤ −2) predicts improved overall survival (Log-rank *p* = 0.00039; HR = 0.88). Survival analysis performed using GEPIA2 with median expression stratification.

Among the selected upregulated genes (*n* = 36), the high-expression group showed a significantly reduced survival probability compared to the low-expression group (Log-rank *p* = 1.3 × 10^−8^; HR = 1.2), indicating a potential association with poor clinical outcomes (Figure 7A).

Conversely, the selected downregulated genes (*n* = 26) displayed the opposite trend: the high-expression group exhibited a better overall survival rate (Log-rank *p* = 0.00039; HR = 0.88), suggesting a potential protective role for these genes when preserved (Figure 7B).

These results reinforce the relevance of HUB genes as potential key regulators of cisplatin-induced neurotoxicity and highlight their value as prognostic indicators in neuro-oncological contexts.

### 2.8. Correlation Analysis of Gene Expression with Immune Cell Infiltration in LGG

We conducted an additional silico analysis to assess the potential association between our differentially expressed HUB genes and immune cell infiltration patterns using the TISIDB portal and the LGG cohort. We focused on three key subtypes: activated CD8+ T cells (Act_CD8), memory B cells (Mem_B), and natural killer (NK) cells.

**Figure 8:**
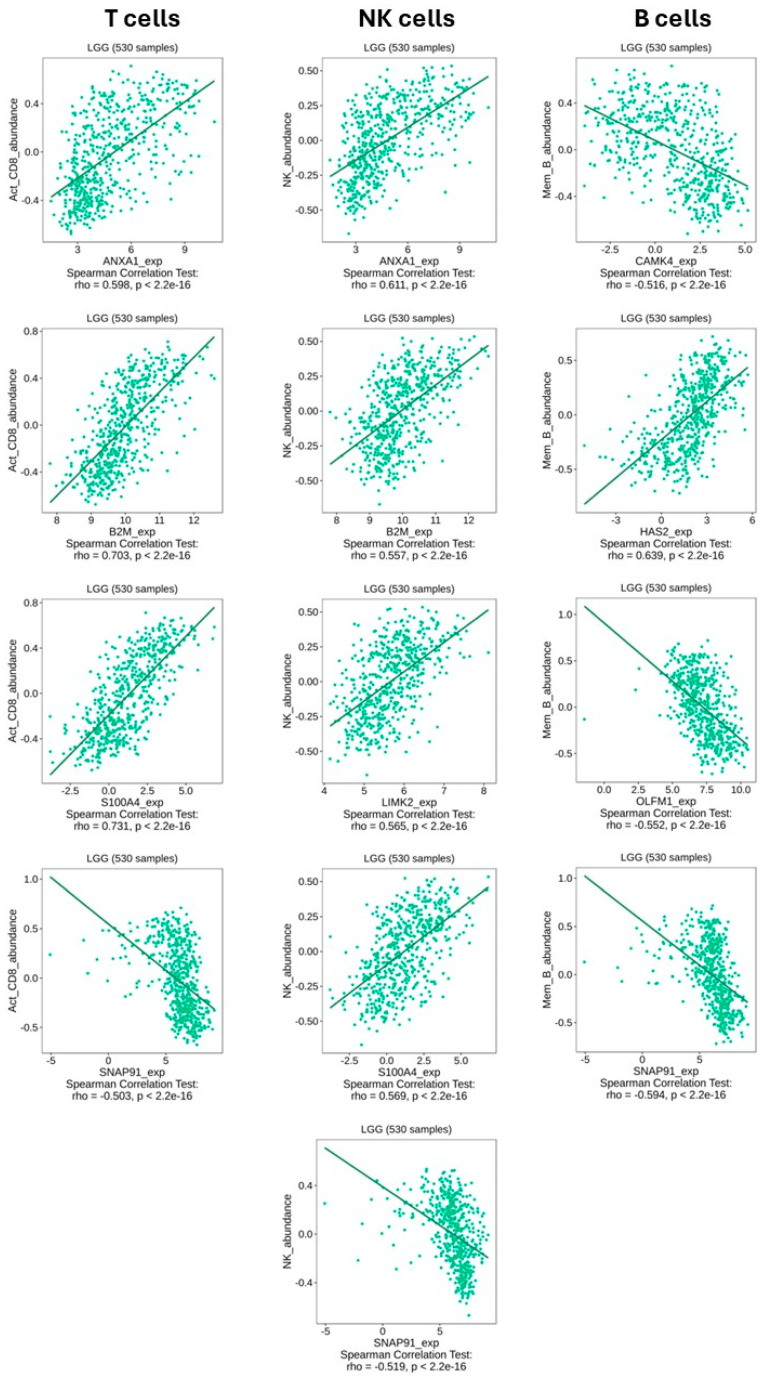
Correlation between upregulated genes and immune cell infiltration in LGG. Scatter plots show Spearman correlation analyses between the expression of selected upregulated genes and the abundance of activated CD8+ T cells (**left column**), NK cells (**middle column**), and memory B cells (**right column**), based on 530 LGG samples from the TISIDB database. All correlations shown are statistically significant (*p* < 2.2 × 10^−16^). Data were obtained from the TISIDB portal (LGG, n = 530).

Among the upregulated HUB genes (Figure 8), several showed significant correlations with immune cell abundance. For instance, *ANXA1* and *B2M* both correlated positively with activated CD8+ T cells (ρ = 0.598 and ρ = 0.703, respectively) and NK cells (ρ = 0.611 and ρ = 0.557, respectively). *S100A4* also showed strong positive correlations with CD8+ T cells (ρ = 0.731) and NK cells (ρ = 0.569), but a negative correlation with memory B cells (ρ = –0.594). In contrast, *SNAP91* showed consistent negative correlations across all three cell types. All reported correlations were highly significant (*p* < 2.2 × 10^−16^).

**Figure 9:**
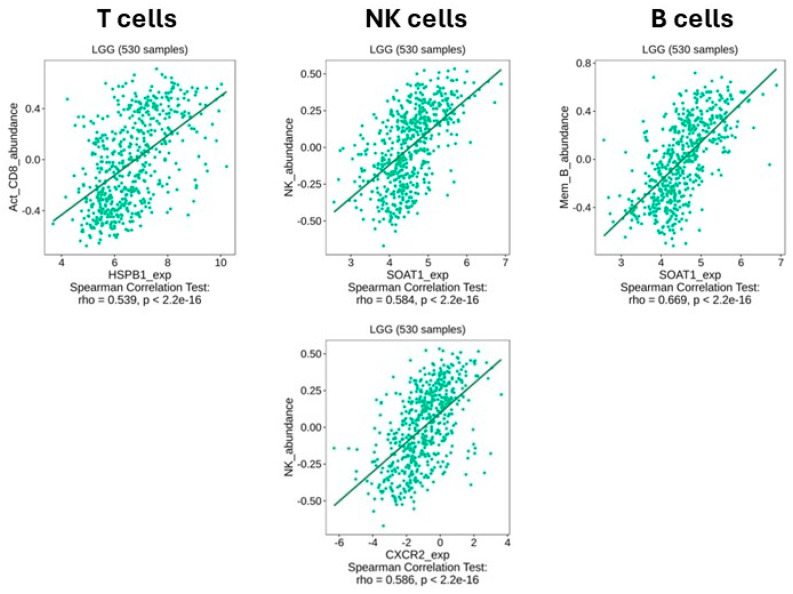
Correlation between downregulated genes and immune cell infiltration in LGG. Each plot illustrates Spearman correlation between the expression of selected downregulated genes and immune cell subtypes: activated CD8+ T cells, NK cells, and memory B cells. Data correspond to 530 LGG tumor samples obtained from the TISIDB platform, with all correlations being statistically significant (*p* < 2.2 × 10^−16^). Data were obtained from the TISIDB portal (LGG, n = 530).

Within the downregulated HUB gene set (Figure 9), we also observed noteworthy associations. *HSPB1* displayed a robust positive correlation with CD8+ T cells (ρ = 0.539, *p* < 2.2 × 10^−16^). Furthermore, *SOAT1* was positively correlated with both NK cells (ρ = 0.584) and memory B cells (ρ = 0.669), while *CXCR2*, a known chemokine receptor, showed a strong positive correlation with NK cells (ρ = 0.586, *p* < 2.2 × 10^−16^).

These findings indicate statistically significant correlations between the expression of cisplatin-regulated genes and the immune landscape within the LGG microenvironment. Although these data are correlational and from a distinct biological context, they suggest that some of the genes affected by cisplatin neurotoxicity may also play roles in immunomodulation, highlighting a potential area for future experimental investigation.

## 3. Discussion

Cisplatin chemotherapy is a cornerstone of cancer treatment, but its efficacy is often limited by severe side effects, including a neurotoxicity syndrome known as chemobrain. This condition, characterized by cognitive deficits, has been linked to synaptic damage, mitochondrial dysfunction, and neuroinflammation. Using a microarray-based approach, this study suggests a transcriptomic characterization of the in vivo effects of a cumulative cisplatin regimen on non-tumoral rat brain tissue. The evidence presented here indicates a complex landscape of altered gene signatures and functional pathways, which may provide novel insights into the molecular underpinnings of cisplatin-induced central nervous system toxicity.

### 3.1. Cisplatin Induces a Unique and Complex Transcriptomic Reprogramming in Brain Tissue

Our analysis revealed a distinct pattern of 143 upregulated and 163 downregulated genes, which showed surprisingly limited overlap with genes previously reported as dysregulated by cisplatin in other biological contexts. This suggests that the brain mounts a unique, tissue-specific transcriptomic response to this chemotherapeutic agent. This novelty highlights the importance of studying cisplatin’s effects directly in intact neural tissue rather than extrapolating from cancer cell lines or peripheral organs.

Despite the overall uniqueness, our comparative analysis highlighted several key genes whose regulation was consistent with prior literature, supporting their central role in the response to cisplatin. Notably, we observed the upregulation of *CAMK4*, *FANCC*, and *GRIA4*. *CAMK4* has been implicated as mediator of CREB-dependent transcription and synaptic plasticity, and its inhibition has been reported to alleviate cisplatin-induced cognitive deficits [15,16,17]. *FANCC* is a core component of the DNA repair machinery responsible for resolving cisplatin-induced interstrand crosslinks, and its proper regulation is crucial for neuronal survival under genotoxic stress [18,19,20]. Finally, *GRIA4*, an AMPA-type glutamate receptor subunit, may point towards an alteration of glutamatergic transmission, which could enhance excitotoxic stress, a known mechanism in chemotherapy-induced neurotoxicity [21,22,23,24]. Similarly, the context-dependent regulation of the cell cycle kinase *CDK4* is particularly relevant, as its aberrant activation in post-mitotic neurons is linked to neurodegenerative processes [25,26].

### 3.2. Dysregulation of Synaptic Signaling Pathways and Neuronal Excitability

A central theme emerging from our pathway and network analyses is a profound dysregulation of synaptic function. Among downregulated genes, the neuroactive ligand–receptor interaction pathway emerged as a suggestively enriched module (FDR ≈ 0.067), pointing to a coordinated suppression of neurotransmitter and neuropeptide receptor signaling.

Concurrently with this suppression, we observed a strong upregulation of pathways related to calcium and neurotrophin signaling pathway. The overexpression of key hubs in our PPI network, such as *PLCG1*, *CAMK4*, and the calcium channel subunit *CACNA1E*, indicates a robust activation of calcium-dependent intracellular cascades. While these pathways are essential for adaptive synaptic plasticity, their sustained and dysregulated activation can also trigger excitotoxic and apoptotic signaling. This dual-edged response, a suppression of baseline neurotransmission alongside a hypersensitive activation of plasticity-related signaling paints a picture of a brain struggling to maintain homeostasis, resulting in an imbalanced and potentially vulnerable state of excitability.

### 3.3. Suppression of Cellular Homeostasis and Neuroprotective Mechanisms

Beyond the synapse, our PPI network analysis of downregulated genes revealed a coordinated suppression of fundamental cellular support systems. We identified significant clusters of downregulated genes involved in mitochondrial function and fatty acid metabolism (e.g., *ACADM*, *SARDH*), consistent with previous reports of ATP depletion and mitochondrial damage following cisplatin administration [27]. This suggests a potential suppression of cerebral energy metabolism, compromising the high energetic demands of the brain.

Furthermore, we observed the downregulation of a critical cluster related to the protein misfolding stress response, including the molecular chaperone *HSPB1*. The repression of such proteostasis machinery could impair the cell’s ability to manage damaged proteins, favoring their accumulation and leading to synaptic dysfunction and neuronal death [28,29,30,31,32,33]. Finally, the downregulation of genes in the CXC chemokine signaling pathway (e.g., *CXCR2*) implies a disruption of neuroimmune communication. This could alter the brain’s ability to mount an effective inflammatory and repair response, potentially leading to sustained damage [34,35,36,37,38,39,40].

### 3.4. Molecular Basis for Chemobrain: Connecting Pathway Dysregulation to Clinical Symptoms

Our findings may offer a functional framework to interpret the complex clinical symptoms of chemobrain from a translational perspective. The enrichment of pathways related to “Amphetamine addiction pathway” and Morphine addiction pathway should not be interpreted literally, but as a powerful indicator of cisplatin’s impact on neural circuits governing reward, motivation, and learning. These pathways share core molecular players—such as *CAMK4*, *PLCG1*, and *GRIA4*—with dopaminergic and glutamatergic signaling in the nucleus accumbens and prefrontal cortex [41,42,43,44,45]. Their dysregulation could contribute to the affective symptoms, anhedonia, and cognitive fatigue reported by patients [1,46,47].

Similarly, the downregulation of genes associated with the Vascular smooth muscle contraction pathway suggests a potential disruption in the autoregulation of cerebral blood flow [48,49]. This could impair neurovascular coupling, a process vital for memory consolidation and sustained attention, possibly helping to explain the deficits in executive function and working memory characteristic of chemobrain [50,51]. These findings should be regarded as hypothesis-generating and invite a critical clinical reflection on designing adjuvant therapies that can preserve the integrity of these cerebral networks without compromising antineoplastic efficacy.

### 3.5. Differentiating Cisplatin Neurotoxicity from Brain Metastasis-Related Symptoms: Clinical and Experimental Considerations

An important clinical challenge is distinguishing cognitive symptoms caused directly by cisplatin neurotoxicity from those arising from brain metastases or tumor-related mechanisms. In our experimental model, we intentionally used non-tumor-bearing rats to isolate the direct neurotoxic effects of cisplatin on healthy brain tissue, thus eliminating confounding variables such as tumor burden, mass effect, blood–brain barrier disruption by metastatic lesions, and tumor-induced neuroinflammation.

Clinically, several features may help differentiate these etiologies:

1. Temporal pattern: Cisplatin-induced cognitive impairment typically develops progressively during or shortly after chemotherapy cycles and may persist long-term [52], whereas metastasis-related symptoms often correlate with tumor progression and may present more acutely with focal neurological deficits [53].
2. Neuroimaging findings: Brain metastases present with contrast-enhancing lesions, mass effect, and perilesional edema on MRI [54], while cisplatin neurotoxicity more commonly shows diffuse white matter changes, hippocampal atrophy, and reduced cerebral blood flow without focal mass lesions [55].
3. Cognitive profile: Chemobrain typically manifests as diffuse deficits in attention, processing speed, and executive function, while metastasis-related symptoms may include more focal deficits depending on lesion location [53].
4. Molecular signatures: Our transcriptomic data suggest that cisplatin induces a distinct pattern of synaptic dysfunction (downregulation of neurotransmitter receptors, dysregulation of calcium signaling) and metabolic suppression that differs from the proliferative, angiogenic, and immune-evasive programs typically observed in brain metastases [56].

From a research perspective, animal models like ours using healthy, tumor-free subjects provide a clean experimental system to dissect the direct neurotoxic mechanisms of cisplatin without the biological noise introduced by tumor-host interactions. Future translational studies could compare transcriptomic profiles from non-tumor-bearing chemotherapy-exposed patients with those from patients with brain metastases to identify biomarker signatures that distinguish these conditions, potentially enabling precision medicine approaches to neuroprotection.

### 3.6. Implications for Neuroprotection and Therapeutic Strategies

Our pathway-level readouts point to practical, testable ways to limit cisplatin-related neurotoxicity without undercutting anticancer benefit. First, the coordinated up-shift in Ca^2+^/calmodulin nodes (*PLCG1*, *CAMK4)* alongside glutamatergic components (*GRIA4*) suggests an excitability-driven component that could be tempered—by moderating CaMK-dependent signaling and carefully dialing back excessive glutamatergic drive to curb calcium overload and downstream stress [57]. Second, the rise in adenosine A2A receptor signaling (*ADORA2A*) highlights selective A2A antagonism as a plausible way to blunt neuroinflammatory and excitatory cascades [58]. Third, enrichment of redox and proteostasis programs (NRF2-linked transcription; HSP chaperone systems) supports strategies that boost endogenous antioxidant capacity and protein quality control (e.g., NRF2 activators or chaperone-supportive interventions) [59]. Fourth, non-pharmacologic options structured aerobic exercise and targeted cognitive training, offer low-risk ways to build resilience against chemotherapy-related cognitive change. Finally, treatment-design choices (schedule refinement, exposure minimization, or nano/liposomal formulations) may help reduce CNS exposure when clinically acceptable [60]. These directions are hypothesis-driven and anchored in our transcriptomic signature; their clinical value will require prospective testing in models that optimize both anti-tumor efficacy and neuroprotection.

### 3.7. Exploratory Correlation with Human Glioma Data: A Hypothesis-Generating Perspective

As a purely exploratory, in silico exercise, we investigated whether the gene signatures identified in our rat neurotoxicity model showed any parallel with clinical outcomes in human lower-grade glioma (LGG). It is crucial to emphasize that this correlational analysis does not imply a causal link, given the profound biological differences between the two contexts. However, the analysis revealed noteworthy parallels. High expression of the upregulated gene signature (including pro-proliferative and stress-resistance genes like *PLCG1* and *CAMK4*) was correlated with poorer patient survival. Conversely, high expression of the downregulated gene signature (including genes involved in homeostatic functions like *CXCR2*) appeared to be associated with better survival.

Similarly, our analysis suggested correlations between cisplatin-regulated genes and the immune landscape of the LGG microenvironment. While this does not provide direct evidence of cisplatin’s immunomodulatory effects in the brain, it highlights that some of the genes affected by cisplatin may also highlights that some genes altered by cisplatin are associated with variation in immune-cell presence estimates in the CNS [61]. These intriguing, though speculative, overlaps suggest that some molecular pathways may be shared between neurotoxicity and neuro-oncology, warranting future experimental investigation.

### 3.8. Novelty and Key Contributions

To our knowledge, comprehensive, whole-brain transcriptomic profiling after a cumulative cisplatin regimen in vivo has been limited. Here, we provide an integrated map of cisplatin-induced transcriptional changes in rat brain under a clinically relevant, multi-cycle dosing schedule and benchmark it against prior literature. Our comparative literature analysis (standardized gene symbols, curated studies up to 2025) underscores limited overlap with previously reported cisplatin-responsive genes across contexts, supporting a brain-specific response profile.

Genome-wide response in whole brain: 306 differentially expressed genes (143 up, 163 down).

Dual pattern of dysregulation: Upregulated genes enrich neural signaling and plasticity programs consistent with calcium/neurotrophin activity, whereas downregulated genes converge on neurotransmitter systems and ion-/metabolic homeostasis modules.

Pathway-level specificity: Significant KEGG pathways include Neurotrophin signaling (rno04722), PPAR (rno03320), Axon guidance (rno04360), among upregulated; and Proximal tubule bicarbonate reclamation (rno04964), Endocrine-regulated calcium reabsorption (rno04961), Purine metabolism (rno00230), among downregulated (with Neuroactive ligand–receptor interaction, rno04080, suggestive, FDR ≈ 0.067).

Network centrality: PPI networks identify central hubs (*PLCG1*, *CAMK4*, *ADORA2A*, *GRIA4*, *CACNA1E*; *HSPB1* within downregulated modules) linking synaptic plasticity and calcium signaling to proteostasis and stress responses.

Translational signal (hypothesis-generating): In silico analyses suggest associations between hub-gene signatures and survival/immune contexture in human lower-grade glioma, emphasizing exploratory relevance rather than causality.

Collectively, these layers reveal that cisplatin exposure activates plasticity-linked calcium/neurotrophin programs while suppressing neuromodulatory receptors and ion/energy-homeostasis pathways, providing a mechanistic scaffold for chemobrain that integrates pathway membership, network centrality, and exploratory clinical correlations.

### 3.9. Limitations of the Study

An important design consideration in our study was the use of non-tumor-bearing rats to isolate the direct neurotoxic effects of cisplatin on healthy brain tissue. This experimental approach eliminates confounding variables such as tumor burden, mass effect, blood–brain barrier disruption by metastatic lesions, and tumor-induced neuroinflammation, which represent a significant clinical challenge when distinguishing cognitive symptoms.

This study has several limitations that should be acknowledged. First, it should be regarded as exploratory. Differential expression was initially defined using Z-score thresholds rather than gene-level FDR, which increases the risk of false positives. Although standard normalization was applied, the reduced final sample size and the lack of independent validation (e.g., qRT-PCR) limit confirmatory claims. Therefore, pathway and PPI inferences are hypothesis-generating and require independent validation. Future studies should prioritize RT-qPCR validation of the top hub genes identified through our network analysis, including *PLCG1*, *CAMK4*, *GRIA4* (upregulated), and *GABRA1*, *GRPR*, and *HSPB1* (downregulated), along with protein-level confirmation via Western blot or immunohistochemistry.

Second, this study used bulk RNA extracted from whole-brain homogenates to obtain an integrated view of cisplatin-induced transcriptional changes. While this approach reduces regional and batch variability and is appropriate for discovery-level analyses, it inherently averages signals across neuronal and glial compartments, potentially attenuating cell-type–specific effects. In this context, pathways related to synaptic transmission, GPCR signaling, and calcium dynamics likely reflect neuronal processes (e.g., activity-dependent excitotoxic responses), whereas shifts in metabolic, redox, and glutamate–glutamine cycle pathways may be driven predominantly by astrocytes. Enrichment of inflammatory and complement cascades is consistent with microglial activation, and changes in myelin/axon-related programs can point to oligodendrocyte involvement. These interpretations remain hypothesis-generating given the bulk design. Future studies will prioritize cell-resolved strategies—such as region-specific dissections, single-nucleus RNA-seq (snRNA-seq), immuno-enrichment/flow sorting (e.g., NeuN^+^ neurons, Olig2^+^ oligodendrocytes, TMEM119^+^ microglia), followed by RNA-seq, or TRAP/RiboTag—to dissect cisplatin responses at cellular resolution and to test whether the bulk-level pathway shifts localize to specific cell populations.

This study was conducted exclusively in male Wistar rats, and the findings may not be generalizable to females, who may exhibit different responses to chemotherapy. Sex differences in neurotoxic responses to cisplatin are biologically plausible, as female rodents and patients often display distinct patterns of drug metabolism, hormonal modulation of neuroinflammation, and resilience to mitochondrial or synaptic stress. Estrogen signaling, in particular, has been shown to influence calcium dynamics, oxidative stress responses, and apoptotic cascades, all of which were highlighted in our transcriptomic analyses [62,63,64]. Thus, it is possible that the pathways and hub genes identified here may behave differently in females, either attenuating or exacerbating cisplatin-induced neurotoxicity. Future studies should incorporate both sexes to determine whether the transcriptomic alterations described are sex-dependent and to better reflect the clinical heterogeneity observed in patients.

Also, the LGG analysis is exploratory and limited by potential confounding inherent to retrospective public cohorts. Although platinum delivery to glioma tissue is pharmacologically plausible under conditions of blood–tumor barrier permeability or local delivery, the current data do not test drug response or causality. Future work should examine whether cisplatin-induced pathways observed in the rat CNS model align with treatment exposure or tumor microenvironmental states in prospective datasets.

## 4. Materials and Methods

### 4.1. Ethical Approval

All experimental procedures were approved by the Animal Care and Use Committee at the Instituto de Investigaciones Cerebrales (IICe) at Universidad Veracruzana, in accordance with the Official Mexican Standard NOM-062-ZOO-1999 (Technical Specifications for the Production, Care and Use of Laboratory Animals). Additionally, experiments adhered to the guidelines established by the U.S. National Institutes of Health (NIH) for animal care and handling.

### 4.2. Animals and Experimental Design

Male Wistar rats (3 months old) were housed under standard vivarium conditions (temperature 22 ± 2 °C, humidity 50 ± 10%, ventilated polycarbonate cages with nesting material enrichment) with Ad Libitum access to food and water. Animals were randomly allocated to a cisplatin-treated group and a saline control group (n = 10 per group). Random sequence was generated by computer and concealed until allocation. Order of handling and measurement was counterbalanced across groups to minimize batch effects. All procedures were conducted under identical laboratory conditions to minimize confounding variables. Cisplatin (cis-diamminedichloroplatinum(II); Sigma-Aldrich (St. Louis, MO, USA); cat. P4394) was dissolved in sterile 0.9% sodium chloride and administered intraperitoneally (i.p.) at 2 mg/kg per injection following a five-week regimen comprising five cycles, each defined as two injections separated by 48 h (cumulative dose = 20 mg/kg). This cumulative dose approximates a human oncology regimen of ~60 mg/m^2^ when scaled by body-surface area [65]. The schedule and dosing were adapted from established models of cisplatin-induced peripheral neuropathy that have also been reported to induce central nervous system alterations [66,67,68]. Control animals received i.p. injections of an equivalent volume of saline on the same schedule.

### 4.3. Animal Care and Monitoring

Animals were acclimatized ≥7 days before procedures and monitored daily for clinical signs, including body weight, general appearance, grooming behavior, activity levels, and food/water consumption. Any signs of distress, significant weight loss (>15% from baseline), abnormal behavior, or other welfare concerns were recorded and evaluated according to predetermined criteria.

Predefined humane endpoints included: severe weight loss (>20% from baseline), persistent lethargy, hunched posture, piloerection, decreased responsiveness to external stimuli, or any signs of severe distress that persisted beyond 24 h despite supportive care. Animals were monitored twice daily (morning and evening) throughout the experimental period, with increased frequency if welfare concerns arose.

Throughout the study period, animals were closely monitored for adverse events related to cisplatin treatment, including nephrotoxicity, gastrointestinal distress, and neurological symptoms. No unexpected serious adverse events occurred during the experiment. Mild, transient weight fluctuations (<10%) were observed in some cisplatin-treated animals but resolved without intervention and did not meet criteria for early termination.

### 4.4. Tissue Collection and RNA Extraction

Following the final treatment cycle, rats were euthanized by CO_2_ inhalation. Whole brains were rapidly extracted; tissues were immediately frozen in liquid nitrogen to ensure RNA integrity and stored at –80 °C for subsequent total RNA extraction.

### 4.5. Total RNA Isolation

Total RNA was isolated from the brain tissues of four biological replicates per group, which were selected based on optimal tissue quality. Tissues were homogenized in 1 mL of TRIzol reagent (Sigma-Aldrich, St. Louis, MO, USA, cat. T9424) according to the manufacturer’s protocol. Briefly, after homogenization, chloroform was added for phase separation, and the samples were centrifuged at 12,000× *g* for 15 min at 4 °C. The upper aqueous phase was collected, and RNA was precipitated with isopropanol. The RNA pellet was then washed with 75% ethanol and rehydrated in RNase-free water. RNA concentration and purity were determined by spectrophotometry using a BioDrop Duo+ (Thermo Scientific, Wilmington, DE, USA), assessing the 260/280 and 260/230 absorbance ratios. RNA integrity was quantified using an Agilent Bioanalyzer 2100, and only samples with an RNA Integrity Number (RIN) ≥ 8.0 were used for subsequent microarray analysis.

### 4.6. Microarray Printing and Slide Preparation

Microarrays were printed using a Rattus norvegicus 70-mer oligonucleotide library (Operon Oligo Sets), consisting of 5000 gene-specific probes. Oligonucleotides were resuspended at 40 µM in Micro Spotting Solution (TeleChem International Inc., Sunnyvale, CA, USA) and printed in duplicate on SuperAmine-coated glass slides (25 × 75 mm) (ArrayIt, Sunnyvale, CA, USA). Slides were fixed at 80 °C for 4 h, rehydrated in water vapor at 60 °C, and UV cross-linked in two cycles (1200 J). After boiling at 92 °C for 2 min, slides were rinsed in 95% ethanol for 1 min and pre-hybridized for 1 h at 42 °C in a solution of 5X SSC, 0.1% SDS, and 1% BSA.

### 4.7. Probe Labeling and Hybridization

For each hybridization, ten micrograms of total RNA from a control sample and a cisplatin-treated sample were used for first-strand cDNA synthesis using the First-Strand cDNA Labeling Kit (Invitrogen, Carlsbad, CA, USA) During synthesis, fluorescent nucleotides dUTP-Alexa555 (for the control group) or dUTP-Alexa647 (for the cisplatin-treated group) were incorporated. The incorporation of fluorophores was evaluated by absorbance measurements. Equal amounts of labeled cDNA were combined, purified, and hybridized to the microarray slides in UniHyb hybridization solution (TeleChem) for 14 h at 42 °C. Slides were subsequently washed three times with 1X SSC and 0.05% SDS at room temperature.

### 4.8. Image Acquisition and Analysis

Microarray images were acquired using a GenePix 4100A scanner with GenePix software Pro 7 (Molecular Devices, San Jose, CA, USA) at 10 µm resolution. For each spot, mean density and background values for Alexa555 and Alexa647 were calculated using ArrayPro Analyzer version 4.5 (Media Cybernetics, Rockville, MD, USA).

### 4.9. Raw Data Analysis

Fluorescence intensity data were analyzed using the open-source software genArise (https://www.bioconductor.org/packages/release/bioc/html/genArise.html, accessed on 20 October 2025) The analysis pipeline included: (1) background correction by subtracting local background from foreground intensity; (2) quality filtering to remove spots with inadequate signal or morphological defects; (3) log-ratio calculation as Log_2_(Cisplatin/Control) = Log_2_(Cy5/Alexa647) − Log_2_(Cy3/Alexa555); (4) LOWESS (locally weighted scatterplot smoothing) normalization with a smoothing parameter of 0.3 to correct for intensity-dependent dye bias; and (5) replicate averaging, where normalized log-ratios for duplicate spots within each array and values from both biological replicate arrays were averaged.

Differentially expressed genes were identified using an intensity-dependent Z-score approach. For each gene, the Z-score was calculated as Z = (R_i_; − μ)/σ, where R_i_; is the normalized log_2_ ratio for gene i, μ is the local mean log_2_ ratio calculated from a sliding window of 500 genes with similar average intensity, and σ is the local standard deviation from the same window. This approach accounts for increased variance at lower expression levels. Genes with an absolute Z-score of |z| ≥ 2 were considered significantly differentially expressed, corresponding approximately to *p* < 0.05. To ensure robust biological interpretation, pathway enrichment analyses (Gene Ontology and KEGG) employed FDR correction (FDR < 0.05), and only pathways with Fold Enrichment ≥ 5 were interpreted. Quality control metrics included RNA integrity (RIN ≥ 8.0), dye-swap consistency between biological replicates (r > 0.85), spot quality (>95% passing filters), and array-to-array reproducibility (r > 0.90).

### 4.10. Comparative Literature Analysis

To evaluate the novelty and context dependence of differentially expressed genes, we conducted a comparative literature analysis. A curated database of previously reported gene expression changes was compiled based on peer-reviewed publications indexed in PubMed and Scopus until January 2005 to January 2025. The search used combinations of keywords such as “cisplatin”, “brain”, “gene expression”, “microarray”, and “neurotoxicity”, and was limited to studies using In Vivo or In Vitro models of cisplatin exposure. Genes were categorized as upregulated, downregulated, or stably expressed under control conditions (SEG), based on explicit Z-score, fold-change, or expression pattern references provided in each publication [12,13]. Only studies with clear experimental conditions (dose, time point, tissue type) and validated gene lists were included. The gene symbols were standardized using the NCBI Gene database to ensure accurate comparison. This dataset was used to generate Venn diagrams and overlap analyses (Figure 1) with our experimental microarray.

### 4.11. Functional Enrichment and Pathway Analysis

To explore the biological functions associated with the differentially expressed genes (DEGs), we performed enrichment analyses for upregulated (Z ≥ 2) and downregulated (Z ≤ −2) gene sets separately.

Pathway enrichment analysis was conducted using ShinyGO: http://bioinformatics.sdstate.edu/go/ (accessed on 15 June 2024). For Gene Ontology (GO) analysis, enrichment was evaluated for three categories: Biological Process (BP), Molecular Function (MF), and Cellular Component (CC). For all analyses, Rattus norvegicus was used as the reference organism with a significance threshold set at a False Discovery Rate (FDR) < 0.05. Only pathways with a Fold Enrichment (FE) ≥ 5 were considered for functional interpretation. To confirm and complement these findings, the DEG lists were also submitted to the DAVID Bioinformatics Resources 6.8 (https://david.ncifcrf.gov/, accessed on 15 June 2024) or complementary functional annotation and clustering analysis.

Multiple testing was controlled using the Benjamini–Hochberg procedure (FDR). Convergence analyses were performed symmetrically for up- and downregulated gene sets; complete KEGG/GO outputs (official pathway name and ID, gene count, Fold Enrichment, and FDR) are provided in Supplementary S1 (significant at FDR ≤ 0.05; suggestive at 0.05–0.10).

### 4.12. Protein–Protein Interaction (PPI) Network Analysis

To evaluate the functional connectivity among DEGs, protein–protein interaction (PPI) networks were constructed using the STRING database (v11.5, https://string-db.org/, accessed on 15 June 2024). Separate analyses were conducted for the upregulated and downregulated gene sets. The analysis was based on high-confidence interaction scores (minimum required interaction score: 0.700), using evidence from curated databases, experimental data, and co-expression. Genes were input using their Rattus norvegicus official gene symbols, and the resulting PPI networks were visualized with nodes representing proteins and edges representing functional associations. From each network, key HUB genes were identified based on a high degree of connectivity (degree ≥ 10), indicating their potential central biological relevance.

### 4.13. Rationale and Scope of the LGG In Silico Contextualization

We performed an In Silico, hypothesis-generating analysis in the TCGA-LGG cohort to examine whether rat-derived cisplatin-responsive signatures show statistical associations with overall survival and immune-cell infiltration estimates. This choice was motivated by mechanistic overlap between cisplatin neurotoxicity (oxidative stress, neuroinflammation, synaptic/mitochondrial dysfunction) and biological programs active in gliomas, including purinergic/A2A signaling; by the documented feasibility of platinum exposure in glioma tissue due to increased blood–tumor barrier permeability and local-delivery approaches; and by the availability of well-annotated public datasets that enable transparent survival modeling and external validation. This analysis is strictly correlational and does not imply causality or therapeutic prediction.

### 4.14. Survival and Immune Infiltration Correlation Analysis

To explore the potential clinical associations of the identified HUB genes, we performed an exploratory, in silico analysis correlating their expression with clinical outcomes in a human dataset. It is important to note that this correlational analysis is hypothesis-generating, linking our findings from a rat neurotoxicity model with publicly available human cancer data.

Survival analysis was performed using the GEPIA2 web server [69] (http://gepia2.cancer-pku.cn/, accessed on 20 February 2025). The expression profiles of HUB genes from our up- and downregulated sets were correlated with overall survival in the lower-grade glioma (LGG) patient cohort from TCGA. Kaplan–Meier survival curves were generated using a median expression cutoff, and the significance was assessed using the log-rank test (*p* ≤ 0.05). Hazard ratios (HR) were also reported.

To investigate potential immunological implications, immune cell infiltration analysis was conducted using the TISIDB platform [70] (http://cis.hku.hk/TISIDB/, accessed on 20 October 2025). We evaluated the Spearman correlation (ρ) between the expression of each HUB gene and the estimated infiltration levels of key immune subsets (activated CD8^+^ T cells, NK cells, and memory B cells) in the LGG cohort, with *p* ≤ 0.05 considered significant.

Complete enrichment outputs are provided in Supplementary S1, including all KEGG/GO terms with the official pathway name and ID, gene count, Fold Enrichment, and FDR (significant: FDR ≤ 0.05; suggestive: 0.05–0.10).

## 5. Conclusions

This study provides a comprehensive transcriptomic map of the molecular changes in the rat brain following a cumulative cisplatin regimen. Our findings reveal a profound genetic imbalance, characterized by the upregulation of adaptive and potentially excitotoxic synaptic signaling pathways alongside a simultaneous downregulation of essential neurotransmitter systems and cellular homeostatic functions. These results offer a rich, hypothesis-generating resource for understanding the molecular basis of chemobrain and identifying numerous candidate targets for neuroprotective strategies. Future studies should aim to validate these transcriptomic signatures, explore their role in the onset and progression of chemotherapy-induced cognitive impairment, and assess the potential of therapeutic interventions that modulate these key pathways to mitigate neurotoxic outcomes.

Future validation studies should employ orthogonal methods, including RT-qPCR, Western blotting, and immunohistochemistry, to confirm the differential expression of candidate genes identified here. Functional assays examining synaptic transmission, calcium dynamics, and mitochondrial function will be essential to establish causal relationships between these transcriptomic changes and cognitive-impairment phenotypes [71,72].

## Figures and Tables

**Table 1:** Selected differentially expressed genes in rat brain tissue following cisplatin treatment.

Gene	Z-Score	Biological Function	Reference
*GSTT1*	4.556	Conjugation of reduced glutathione to a wide number of exogenous and endogenous hydrophobic electrophiles. Acts on 1,2-epoxy-3-(4-nitrophenoxy)propane, phenethylisothiocyanate, 4-nitrobenzyl chloride, and 4-nitrophenethyl bromide. Displays glutathione peroxidase activity with cumene hydroperoxide.	GeneCards-GSTT1
*ENTPD6*	3.931	Catalyzes the hydrolysis of nucleoside triphosphates and diphosphates in a calcium- or magnesium-dependent manner. Has a strong preference for nucleoside diphosphates, preferentially hydrolyzes GDP, IDP, and UDP, with slower hydrolysis of CDP, ITP, GTP, CTP, ADP, and UTP, and virtually no hydrolysis of ATP.	GeneCards-ENTPD6
*KMO*	3.67	Catalyzes the hydroxylation of L-kynurenine (L-Kyn) to form 3-hydroxy-L-kynurenine (L-3OHKyn).	GeneCards-KMO
*GRIA4*	3.312	An ionotropic glutamate receptor that functions as a ligand-gated cation channel, gated by L-glutamate and glutamatergic agonists such as alpha-amino-3-hydroxy-5-methyl-4-isoxazolepropionic acid (AMPA), quisqualic acid, and kainic acid (By similarity). L-glutamate acts as an excitatory neurotransmitter at many synapses in the central nervous system and plays an important role in fast excitatory synaptic transmission (By similarity).	GeneCards-GRIA4
*PTPN11*	3.097	Acts downstream of various receptors and cytoplasmic protein tyrosine kinases to participate in the signal transduction from the cell surface to the nucleus.	GeneCards-PTPN11
*ADORA2A*	2.88	Receptor for adenosine (By similarity). The activity of this receptor is mediated by G proteins, which activate adenylyl cyclase (By similarity).	GeneCards-ADORA2A
*TACR1*	2.879	This is a receptor for the tachykinin neuropeptide substance P. It is probably associated with G proteins that activate a phosphatidylinositol-calcium second messenger system.	GeneCards-TACR1
*ATP2B3*	2.816	ATP-driven Ca^2+^ ion pump involved in the maintenance of basal intracellular Ca^2+^ levels at the presynaptic terminals. Uses ATP as an energy source to transport cytosolic Ca^2+^ ions across the plasma membrane to the extracellular compartment.	GeneCards-ATP2B3
*DDC*	2.768	Catalyzes the decarboxylation of L-3,4-dihydroxyphenylalanine (DOPA) to dopamine and L-5-hydroxytryptophan to serotonin.	GeneCards-DDC
*SYNJ2*	2.762	Inositol 5-phosphatase, which may be involved in distinct membrane trafficking and signal transduction pathways. It may mediate the inhibitory effect of Rac1 on endocytosis.	GeneCards-SYNJ2
*PLCG1*	2.643	Mediates the production of the second messenger molecules diacylglycerol (DAG) and inositol 1,4,5-trisphosphate (IP3). Plays an important role in the regulation of intracellular signaling cascades. Becomes activated in response to ligand-mediated activation of receptor-type tyrosine kinases, such as *Pdgfra*, *Pdgfrb*, *Egfr*, *Fgfr1*, *Fgfr2*, *Fgfr3*, and *Fgfr4* (By similarity). Plays a role in actin reorganization and cell migration.	GeneCards-PLCG1
*PDE4D*	2.62	Hydrolyzes the second messenger cAMP, which is a key regulator of many important physiological processes.	GeneCards-PDE4D
*NFE2L2*	2.596	A transcription factor that plays a key role in the response to oxidative stress: binds to antioxidant response (ARE) elements present in the promoter region of many cytoprotective genes, such as phase 2 detoxifying enzymes, and promotes their expression, thereby neutralizing reactive electrophiles.	GeneCards-NFE2L2
*SORT1*	2.497	Functions as a sorting receptor in the Golgi compartment and as a clearance receptor on the cell surface. Required for protein transport from the Golgi apparatus to the lysosomes by a pathway that is independent of the mannose-6-phosphate receptor (M6PR).	GeneCards-SORT1
*C1GALT1*	2.463	Glycosyltransferase that generates the core 1 O-glycan Gal-beta1-3GalNAc-alpha1-Ser/Thr (T antigen), which is a precursor for many extended O-glycans in glycoproteins.	GeneCards-C1GALT1
*PNLIP*	2.361	plays an important role in fat metabolism. It preferentially splits the esters of long-chain fatty acids at positions 1 and 3, producing mainly 2-monoacylglycerol and free fatty acids, and shows considerably higher activity against insoluble emulsified substrates than against soluble ones.	GeneCards-PNLIP
*ALAS1*	2.271	Catalyzes the pyridoxal 5′-phosphate (PLP)-dependent condensation of succinyl-CoA and glycine to form aminolevulinic acid (ALA), with CoA and CO_2_ as by-products.	GeneCards-ALAS1
*CDK4*	2.254	Ser/Thr-kinase component of cyclin D-CDK4 (DC) complexes that phosphorylate and inhibit members of the retinoblastoma (RB) protein family, including RB1, and regulate the cell-cycle during G(1)/S transition.	GeneCards-CDK4
*CYCTP*	2.215	Predicted to be involved in mitochondrial electron transport, cytochrome c to oxygen, and mitochondrial electron transport, ubiquinol to cytochrome c. Predicted to act upstream of or within the hydrogen peroxide metabolic process and positive regulation of the intrinsic apoptotic signaling pathway.	GeneCards-CYCT
*PFKFB2*	2.21	Synthesis and degradation of fructose 2,6-bisphosphate.	GeneCards-PFKFB2
*GAB2*	2.144	An adapter protein which acts downstream of several membrane receptors, including cytokine, antigen, hormone, cell–matrix and growth factor receptors to regulate multiple signaling pathways. Regulates osteoclast differentiation, mediating the TNFRSF11A/RANK signaling. In allergic response, it plays a role in mast cells activation and degranulation through PI-3-kinase regulation. Also involved in the regulation of cell proliferation and hematopoiesis.	GeneCards-GAB2
*CAMK4*	2.128	Calcium/calmodulin-dependent protein kinase that operates in the calcium-triggered *Camkk-Camk4* signaling cascade and regulates, mainly by phosphorylation, the activity of several transcription activators, such as *Creb1*, *Mef2d*, *Jun* and *Rora*, which play pivotal roles in immune response, inflammation, and memory consolidation.	GeneCards-CAMK4
*HAL*	2.11	The histidine ammonia lyase (histidase),histidine catabolism.	GeneCards-HAL
*CACNA1E*	2.107	Voltage-sensitive calcium channels (VSCC) mediate the entry of calcium ions into excitable cells. They are also involved in a variety of calcium-dependent processes, including muscle contraction, hormone or neurotransmitter release, gene expression, cell motility, cell division, and cell death.	GeneCards-CACNA1E
*CYP7A1*	2.019	Functions as a critical regulatory enzyme of bile acid biosynthesis and cholesterol homeostasis. Catalyzes the hydroxylation of carbon hydrogen bond at the 7-alpha position of cholesterol, a rate-limiting step in cholesterol catabolism and bile acid biosynthesis.	GeneCards-CYP7A1
*GLS*	−2.017	Catalyzes the first reaction in the primary pathway for the renal catabolism of glutamine. Plays a role in maintaining acid-base homeostasis. Regulates the levels of the neurotransmitter glutamate, the main excitatory neurotransmitter in the brain.	GeneCards-GLS
*PLA2G2A*	−2.018	Secretory calcium-dependent phospholipase A2 that primarily targets extracellular phospholipids with implications in host antimicrobial defense, inflammatory response, and tissue regeneration.	GeneCards-PLA2G2A
*SARDH*	−2.032	Catalyzes the last step of the oxidative degradation of choline to glycine. Converts sarcosine into glycine.	GeneCards-SARDH
*PRKCH*	−2.049	Calcium-independent, phospholipid- and diacylglycerol (DAG)-dependent serine/threonine-protein kinase that is involved in the regulation of cell differentiation in keratinocytes and pre-B cell receptor, mediates regulation of epithelial tight junction integrity and foam cell formation, and is required for glioblastoma proliferation and apoptosis prevention in MCF-7 cells.	GeneCards-PRKCH
*SMARCD2*	−2.083	Involved in transcriptional activation and repression of select genes by chromatin remodeling (alteration of DNA-nucleosome topology). Component of SWI/SNF chromatin remodeling complexes that carry out key enzymatic activities, changing chromatin structure by altering DNA-histone contacts within a nucleosome in an ATP-dependent manner.	GeneCards-SMARCD2
*ATP1A2*	−2.133	This is the catalytic component of the active enzyme, which catalyzes the hydrolysis of ATP coupled with the exchange of sodium and potassium ions across the plasma membrane.	GeneCards-ATP1A2
*SHH*	−2.137	The C-terminal part of the sonic hedgehog protein precursor displays an autoproteolysis and a cholesterol transferase activity (By similarity). Both activities result in the cleavage of the full-length protein into two parts (ShhN and ShhC), followed by the covalent attachment of a cholesterol moiety to the C-terminal of the newly generated ShhN (By similarity). Both activities occur in the endoplasmic reticulum (By similarity).	GeneCards-SHH
*GABRR1*	−2.139	The Rho subunit of the pentameric ligand-gated chloride channels is responsible for mediating the effects of gamma-aminobutyric acid (GABA), the major inhibitory neurotransmitter in the brain.	GeneCards-GABRR1
*FZD9*	−2.141	Receptor for WNT2 that is coupled to the beta-catenin canonical signaling pathway, which leads to the activation of disheveled proteins, inhibition of GSK-3 kinase, nuclear accumulation of beta-catenin, and activation of Wnt target genes (By similarity). Plays a role in neuromuscular junction (NMJ) assembly by negatively regulating the clustering of acetylcholine receptors (AChR) through the beta-catenin canonical signaling pathway (By similarity).	GeneCards-FZD9
*ACADM*	−2.2	Medium-chain specific acyl-CoA dehydrogenase is one of the acyl-CoA dehydrogenases that catalyze the first step of mitochondrial fatty acid beta-oxidation, an aerobic process that breaks down fatty acids into acetyl-CoA and allowing the production of energy from fats.	GeneCards-ACADM
*TRH*	−2.206	As a component of the hypothalamic-pituitary-thyroid axis, it controls the secretion of thyroid-stimulating hormone (TSH) and is involved in thyroid hormone synthesis regulation. It also operates as a modulator of hair growth.	GeneCards-TRH
*RALA*	−2.215	Multifunctional GTPase involved in a variety of cellular processes, including gene expression, cell migration, cell proliferation, oncogenic transformation, and membrane trafficking.	GeneCards-RALA
*AP2S1*	−2.235	Component of the adaptor protein complex 2 (AP-2). Adaptor protein complexes function in protein transport via transport vesicles in different membrane traffic pathways. Adaptor protein complexes are vesicle coat components and appear to be involved in cargo selection and vesicle formation.	GeneCards-AP2S1
*CHRNE*	−2.236	After binding acetylcholine, the AChR responds by an extensive change in conformation that affects all subunits and leads to the opening of an ion-conducting channel across the plasma membrane.	GeneCards-CHRNE
*ADORA1*	−2.301	Receptor for adenosine. The activity of this receptor is mediated by G proteins, which inhibit adenylyl cyclase.	GeneCards-ADORA1
*PRKCZ*	−2.307	Calcium- and diacylglycerol-independent serine/threonine-protein kinase that functions in phosphatidylinositol 3-kinase (PI3K) pathway and mitogen-activated protein (MAP) kinase cascade, and is involved in NF-kappa-B activation, mitogenic signaling, cell proliferation, cell polarity, inflammatory response, and maintenance of long-term potentiation (LTP).	GeneCards-PRKCZ
*RASGRP4*	−2.314	Functions as a cation- and diacylglycerol (DAG)-regulated nucleotide exchange factor activating Ras through the exchange of bound GDP for GTP,	GeneCards-RASGRP4
*NPPB*	−2.337	[Brain natriuretic peptide 32]: Cardiac hormone that plays a key role in mediating cardio-renal homeostasis. May also function as a paracrine antifibrotic factor in the heart (By similarity).	GeneCards-NPPB
*PAH*	−2.349	Catalyzes the hydroxylation of L-phenylalanine to L-tyrosine.	GeneCards-PAH
*PLCB4*	−2.352	Activated phosphatidylinositol-specific phospholipase C enzymes catalyze the production of the second messenger molecules diacylglycerol (DAG) and inositol 1,4,5-trisphosphate (IP3) involved in G-protein coupled receptor signaling pathways.	GeneCards-PLCB4
*EDN2*	−2.412	Endothelins are endothelium-derived vasoconstrictor peptides.	GeneCards-EDN2
*RPS6KB1*	−2.473	Serine/threonine-protein kinase that acts downstream of mTOR signaling in response to growth factors and nutrients to promote cell proliferation, cell growth, and cell cycle progression.	GeneCards-RPS6KB1
*FGF10*	−2.551	Plays an important role in the regulation of embryonic development, cell proliferation, and cell differentiation. Required for normal branching morphogenesis. It may play a role in wound healing.	GeneCards-FGF10
*PDE7A*	−2.689	Hydrolyzes the second messenger cAMP, which is a key regulator of many important physiological processes.	GeneCards-PDE7A
*PDE1C*	−2.772	Calmodulin-dependent cyclic nucleotide phosphodiesterase with a dual specificity for the second messengers cAMP and cGMP, which are key regulators of many important physiological processes.	GeneCards-PDE1C
*ODC1*	−2.864	Catalyzes the first and rate-limiting step of polyamine biosynthesis that converts ornithine into putrescine, which is the precursor for the polyamines, spermidine and spermine. Polyamines are essential for cell proliferation and are implicated in cellular processes, ranging from DNA replication to apoptosis.	GeneCards-ODC1
*XDH*	−2.948	Key enzyme in purine degradation. Catalyzes the oxidation of hypoxanthine to xanthine. Catalyzes the oxidation of xanthine to uric acid. Contributes to the generation of reactive oxygen species. Also has low oxidase activity towards aldehydes (in vitro).	GeneCards-XDH
*RLN1*	−3.042	Relaxin is an ovarian hormone that acts with estrogen to produce dilatation of the birth canal in many mammals. May be involved in remodeling of connective tissues during pregnancy, promoting growth of pubic ligaments and ripening of the cervix.	GeneCards-RLN1
*MVD*	−3.1	Catalyzes the ATP-dependent decarboxylation of (R)-5-diphosphomevalonate to form isopentenyl diphosphate (IPP). Functions in the mevalonate (MVA) pathway leading to isopentenyl diphosphate (IPP), a key precursor for the biosynthesis of isoprenoids and sterol synthesis.	GeneCards-MVD
*GRPR*	−3.15	Receptor for gastrin-releasing peptide (GRP). Signals via association with G proteins activate a phosphatidylinositol-calcium second messenger system, resulting in Akt phosphorylation. Contributes to the regulation of food intake. Contributes to the perception of prurient stimuli and transmission of itch signals in the spinal cord that promote scratching behavior, but does not play a role in the perception of pain.	GeneCards-GRPR
*HMBS*	−3.406	As part of the heme biosynthetic pathway, it catalyzes the sequential polymerization of four molecules of porphobilinogen to form hydroxymethylbilane, also known as preuroporphyrinogen.	GeneCards-HMBS

Genes are ordered by Z-score. The table displays the top 25 upregulated and top 30 downregulated genes identified in the microarray analysis. Biological functions were curated from the GeneCards database (www.genecards.org, 5 January 20025). Supplementary Information: added Table S1. Extended gene list with neuro function/neurotoxicity relevance and cross-referenced it in the main text.

## Data Availability

The datasets generated and analyzed during the current study are available from the corresponding author upon reasonable request. Due to ethical and privacy considerations involving animal subjects and proprietary microarray data processing pipelines, data are not publicly available.

## Supplementary Materials

The following supporting information can be downloaded at: https://www.mdpi.com/article/10.3390/ijms262110299/s1.

## Abbreviations

The following abbreviations are used in this manuscript:

ACADM	Acyl-CoA Dehydrogenase Medium Chain
AKT	AKT Serine/Threonine Kinase (Protein Kinase B)
AMPA	α-amino-3-hydroxy-5-methyl-4-isoxazolepropionic acid receptor
AMPK	AMP-Activated Protein Kinase
APC	Adenomatous Polyposis Coli Protein
BAX	Bcl-2-associated X protein (pro-apoptotic)
BCL2	B-cell lymphoma 2 protein (anti-apoptotic)
BDNF	Brain-Derived Neurotrophic Factor
BP	Biological Process (Gene Ontology term)
CAMK2A	Calcium/Calmodulin-Dependent Protein Kinase II Alpha
CAMK4	Calcium/Calmodulin-Dependent Protein Kinase IV
CAMP	Cyclic Adenosine Monophosphate
CASP8	Caspase-8
Caspase-3	Cysteine-aspartic acid protease 3
CC	Cellular Component (Gene Ontology term)
CD8+	Cytotoxic T lymphocytes
CDDP	Cisplatin
CDK12	Cyclin-Dependent Kinase 12
CDK13	Cyclin-Dependent Kinase 13
CDK4	Cyclin-Dependent Kinase 4
CDKN1A	Cyclin-Dependent Kinase Inhibitor 1A
cDNA	Complementary DNA
CHRM1	Muscarinic Acetylcholine Receptor M1
CNS	Central Nervous System
CREB	cAMP Response Element-Binding Protein
CXCL2	C-X-C Motif Chemokine Ligand 2
CXCR2	C-X-C Motif Chemokine Receptor 2
CytoHubba 0.1	Cytoscape plugin for identifying hub genes in networks
Cytoscape 3.10.4	Cytoscape Network Visualization Software
DEGs	Differentially Expressed Genes
DEPC	Diethylpyrocarbonate
DNA	Deoxyribonucleic acid
GABA	Gamma-Aminobutyric Acid (inhibitory neurotransmitter)
GABAA	GABA type A receptor
GABAergic synapse	Synaptic signaling via gamma-aminobutyric acid
GABRA1	Gamma-Aminobutyric Acid Receptor Subunit Alpha-1
GABRR1	Gamma-Aminobutyric Acid Receptor Subunit Rho-1
genArise	Gene Expression Analysis Software (UNAM)
GEPIA2	Gene Expression Profiling Interactive Analysis 2
GFAP	Glial Fibrillary Acidic Protein
GO	Gene Ontology
GPCR	G Protein-Coupled Receptors
GRIA1	Glutamate Ionotropic Receptor AMPA Type Subunit 1
GRIA4	Glutamate Ionotropic Receptor AMPA Type Subunit 4
GRK5	G Protein-Coupled Receptor Kinase 5
GRPR	Gastrin-Releasing Peptide Receptor
HDAC6	Histone Deacetylase 6
HSPB1	Heat Shock Protein Beta-1
i.p.	Intraperitoneal
IL8	Interleukin 8
JAK	Janus Kinase
KEGG	Kyoto Encyclopedia of Genes and Genomes
LGG	Low-Grade Glioma
MAPK	Mitogen-Activated Protein Kinase
MAPK1	Mitogen-Activated Protein Kinase 1
MAPK14	Mitogen-Activated Protein Kinase 14
MAPK3	Mitogen-Activated Protein Kinase 3
MCODE	Molecular Complex Detection (Cytoscape plugin)
Memory B cells	Memory B lymphocytes
MF	Molecular Function (Gene Ontology term)
mRNA	Messenger RNA
MTOR	Mechanistic Target of Rapamycin
NF-κB	Nuclear Factor kappa-light-chain-enhancer of activated B cells
NGF	Nerve Growth Factor
NK	Natural Killer cells
NMDA	N-Methyl-D-Aspartate
Nrf2	Nuclear factor erythroid 2-related factor 2
OD	Optical Density
p53	Tumor suppressor protein p53
PBS	Phosphate-Buffered Saline
PLA2G2A	Phospholipase A2 Group IIA
PLCB1	Phospholipase C Beta 1
PLCB4	Phospholipase C Beta 4
PLCG1	Phospholipase C Gamma 1
PPAR	Peroxisome Proliferator-Activated Receptor family/pathway
PPI	Protein–Protein Interaction
PRKCZ	Protein Kinase C Zeta
PTPN11	Protein Tyrosine Phosphatase Non-Receptor Type 11
qRT-PCR	Quantitative Real-Time Polymerase Chain Reaction
Rap1	Ras-proximate-1
RNA	Ribonucleic acid
RXRA	Retinoid X Receptor Alpha
SARDH	Sarcosine Dehydrogenase
SH2B1	SH2B Adaptor Protein 1
ShinyGO	Shiny Gene Ontology Enrichment Tool
SORT1	Sortilin 1
STAT	Signal Transducer and Activator of Transcription family
STRING	Search Tool for the Retrieval of Interacting Genes/Proteins
TISIDB	Tumor and Immune System Interaction Database
TRHR	Thyrotropin-Releasing Hormone Receptor
TRIzol	Commercial reagent for RNA isolation
VEGF	Vascular Endothelial Growth Factor
Z-score	Standard score (Z-score)
