# Supplementary material for: Exploratory Gene Expression Profiling of Cisplatin-Induced Neurotoxicity in Rat Brain
Source: Int J Mol Sci. 2025 Oct 23;26(21):10299. doi: 10.3390/ijms262110299 (PMC12609972; doi:10.3390/ijms262110299)
Supplement: Supplementary file 1 [file ijms-26-10299-s001.zip › S1.pdf]

Functional Enrichment and Pathway Analysis:

*Full enrichment outputs are reported in **Supplementary Table S1**, including all KEGG/GO terms with pathway name, official ID, gene count, fold enrichment, and FDR (significant: FDR ≤ 0.05; suggestive: 0.05–0.10).*

Upregulated (FDR ≤ 0.05)

- Amphetamine addiction (rno05031) — 4 genes, FDR 0.0094
- PPAR signaling pathway (rno03320) — 4 genes, FDR 0.0100
- Fc gamma R–mediated phagocytosis (rno04666) — 4 genes, FDR 0.0210
- Neurotrophin signaling pathway (rno04722) — 4 genes, FDR 0.0320
- Axon guidance (rno04360) — 5 genes, FDR 0.0220
- Human immunodeficiency virus 1 infection (rno05170) — 5 genes, FDR 0.0320
- cAMP signaling pathway (rno04024) — 5 genes, FDR 0.0390
- Metabolic pathways (rno01100) — 17 genes, FDR 0.0081

Downregulated (FDR ≤ 0.05)

- Proximal tubule bicarbonate reclamation (rno04964) — 2 genes, FDR 0.046
- Hepatitis B (rno05161) — 3 genes, FDR 0.024
- Endocrine and other factor-regulated calcium reabsorption (rno04961) — 3 genes, FDR 0.025
- Morphine addiction (rno05032) — 4 genes, FDR 0.014
- Purine metabolism (rno00230) — 4 genes, FDR 0.029
- Gastric cancer (rno05226) — 4 genes, FDR 0.044
- cGMP–PKG signaling pathway (rno04022) — 4 genes, FDR 0.046
- Pathways in cancer (rno05200) — 7 genes, FDR 0.046
- Metabolic pathways (rno01100) — 13 genes, FDR 0.046

Suggestive (0.05 < FDR ≤ 0.10)

- Renin secretion (rno04924) — 4 genes, FDR 0.067
- Vascular smooth muscle contraction (rno04270) — 5 genes, FDR 0.067
- Neuroactive ligand–receptor interaction (rno04080) — 8 genes, FDR 0.067
