# Supplementary material for: Exploratory Gene Expression Profiling of Cisplatin-Induced Neurotoxicity in Rat Brain
Source: Int J Mol Sci. 2025 Oct 23;26(21):10299. doi: 10.3390/ijms262110299 (PMC12609972; doi:10.3390/ijms262110299)
Supplement: Supplementary file 1 [file ijms-26-10299-s001.zip › Table S1.pdf]

Table S1. Extended gene list with neuro function / neurotoxicity relevance

Gene	Z-score	General biological function	Neuro function / relevance to neurotoxicity
**PLCG1**	2.643	Phospholipase C gamma 1; catalyzes PIP2 → IP3 + DAG signaling.	Links receptor activation to Ca^{2+} release; amplifies Ca^{2+} transients driving activity-dependent stress and synaptic remodeling.
**CAMK4**	2.128	Calcium/calmodulin-dependent protein kinase IV; nuclear signaling to CREB.	Regulates plasticity/memory gene programs; overactivation may worsen Ca^{2+}-driven injury under chemotherapy.
**ADORA2A**	2.88	Adenosine A2A GPCR; modulates cAMP and inflammatory cascades.	Shapes cortico-striatal excitability and neuroinflammation; antagonism is a plausible neuroprotective lever.
**GRIA4**	3.312	Glutamate AMPA receptor subunit; fast excitatory transmission.	Excessive AMPA signaling can promote excitotoxic stress and cognitive dysfunction.
**ATP2B3**	2.816	Plasma membrane Ca^{2+}-ATPase (PMCA3); Ca^{2+} extrusion.	Maintains presynaptic Ca^{2+} homeostasis; dysfunction heightens vulnerability to Ca^{2+} overload.
**DDC**	2.768	Aromatic L-amino acid decarboxylase; dopamine/serotonin synthesis.	Alters monoaminergic tone relevant to cognition and affect during chemobrain.
**SYNJ2**	2.762	Phosphoinositide phosphatase;	Controls synaptic vesicle recycling and

		endocytosis and membrane trafficking.	receptor trafficking; perturbation impacts synaptic maintenance.
**PDE4D**	2.62	cAMP-specific phosphodiesterase; terminates cAMP signaling.	Balances cAMP–PKA–CREB plasticity pathways; dysregulation may impair learning/memory.
**NFE2L2**	2.596	Transcription factor NRF2; antioxidant and cytoprotective response.	Compensatory defense against cisplatin-induced oxidative stress; therapeutic target for neuroprotection.
**SORT1**	2.497	Sorting receptor; Golgi/lysosomal protein trafficking and neurotrophin processing.	Influences axonal transport and trophic signaling; disruption reduces neuronal resilience.
**GABRR1**	-2.139	GABA-A rho subunit; inhibitory neurotransmission (retina/cortex).	Down-regulation suggests weakened inhibition and potential hyperexcitability under stress.
**FZD9**	-2.141	Frizzled-9 WNT receptor; development and synaptic maintenance.	Reduction may compromise plasticity and recovery after injury/toxic insult.
**ACADM**	-2.2	Medium-chain acyl-CoA dehydrogenase; mitochondrial β-oxidation.	Energy supply for neurons/astrocytes; decreased expression worsens energetic stress with chemotherapy.
**TRH**	-2.206	Thyrotropin-releasing hormone; neuropeptide signaling.	Neuromodulation of arousal and cognition; reduction may contribute to cognitive/affective symptoms.
